# Unique Regulation of Enterocyte Brush Border Membrane Na-Glutamine and Na-Alanine Co-Transport by Peroxynitrite during Chronic Intestinal Inflammation

**DOI:** 10.3390/ijms20061504

**Published:** 2019-03-26

**Authors:** Subha Arthur, Palanikumar Manoharan, Shanmuga Sundaram, M Motiur Rahman, Balasubramanian Palaniappan, Uma Sundaram

**Affiliations:** 1 Department of Clinical and Translational Sciences and Appalachian Clinical and Translational Science Institute, Joan C. Edwards School of Medicine, Marshall University, 1600 Medical Center Drive, Huntington, WV 25701, USA; arthursu@marshall.edu (S.A.); sundaram1@live.marshall.edu (S.S.); rahmanmd@marshall.edu (M.M.R.); Palaniappan@marshall.edu (B.P.); 2 Department of Molecular Genetics, Biochemistry & Microbiology, University of Cincinnati, Cincinnati, OH 45221, USA; girimicro@yahoo.com

**Keywords:** peroxynitrite, chronic intestinal inflammation, Na-amino acid co-transport, ASCT1, B0AT1

## Abstract

Na-amino acid co-transporters (NaAAcT) are uniquely affected in rabbit intestinal villus cell brush border membrane (BBM) during chronic intestinal inflammation. Specifically, Na-alanine co-transport (ASCT1) is inhibited secondary to a reduction in the affinity of the co-transporter for alanine, whereas Na-glutamine co-transport (B0AT1) is inhibited secondary to a reduction in BBM co-transporter numbers. During chronic intestinal inflammation, there is abundant production of the potent oxidant peroxynitrite (OONO). However, whether OONO mediates the unique alteration in NaAAcT in intestinal epithelial cells during chronic intestinal inflammation is unknown. In this study, ASCT1 and B0AT1 were inhibited by OONO in vitro. The mechanism of inhibition of ASCT1 by OONO was secondary to a reduction in the affinity of the co-transporter for alanine, and secondary to a reduction in the number of co-transporters for B0AT1, which were further confirmed by Western blot analyses. In conclusion, peroxynitrite inhibited both BBM ASCT1 and B0AT1 in intestinal epithelial cells but by different mechanisms. These alterations in the villus cells are similar to those seen in the rabbit model of chronic enteritis. Therefore, this study indicates that peroxynitrite may mediate the inhibition of ASCT1 and B0AT1 during inflammation, when OONO levels are known to be elevated in the mucosa.

## 1. Introduction

Amino acids are the essential building blocks of protein synthesis. They are well absorbed from the mammalian small intestine primarily via Na-dependent amino acid co-transporter systems. These secondary active co-transporters are present in the brush border membrane (BBM) of enterocytes [1]. The favorable Na-gradient for these co-transporters is provided by Na/K-ATPase on the basolateral membrane of intestinal epithelial cells.

Glutamine, the most abundant amino acid, is the principal nutrient for the intestinal epithelium. In addition, glutamine plays a vital role in maintaining the mucosal integrity and health in gastrointestinal disorders [1,2]. It is primarily absorbed via the Na-dependent glutamine co-transporter B0AT1 (SLC6A19) expressed on the BBM of villus cells in rabbit small intestine and in rat small intestine-derived epithelial cell line IEC-18 [3,4]. The neutral amino acid alanine is predominantly assimilated by Na-alanine co-transporter, ATB0 (SLC6A14), in the BBM of villus cells in a rabbit small intestine and by alanine, serine, and cysteine transporter 1 (ASCT1, SLC1A4) in IEC-18 cells.

In human inflammatory bowel disease (IBD), such as chronic enteritis, the malabsorption of water, nutrients, and electrolytes leads to the most disabling morbidities of the gastrointestinal disease, namely, malnutrition and diarrhea [1,2,3,4,5,6]. In a rabbit model of chronic enteritis that resembles human IBD, the inhibition of coupled Na-glucose co-transport and NaCl absorption in the small intestine has been described previously [4,6]. Furthermore, in this animal model, the regulation of Na-dependent neutral amino acid transporter systems for glutamine and alanine were also profoundly and uniquely affected. B0AT1 was inhibited during chronic intestinal inflammation in the villus cells [7]. The mechanism of inhibition of B0AT1 was secondary to a reduction in the number of BBM co-transporters without a change in the affinity of the co-transporter for glutamine. In this model, System ATB0, which has been shown to be the predominant alanine cotransporter in the BBM of villus cells, was also inhibited [8]. However, the mechanism of inhibition was secondary to a decrease in the affinity of the co-transporter ATB0 for alanine, without a change in the number of BBM co-transporters. Additionally, treatment with a broad-spectrum immune modulator such as methylprednisolone reversed the inhibition of B0AT1 and ATB0 activities [8,9]. This indicated the involvement of immune inflammatory mediators in the regulation of these co-transporters during chronic intestinal inflammation. 

Inducible nitric oxide (iNO) levels are known to be elevated in the inflamed mucosa due to increased activity of iNO synthase (iNOS) [10,11]. Similarly, the levels of superoxide, a reactive metabolite of oxygen, is also known to be increased in inflamed mucosa [12]. Inducible nitric oxide is known to react with the superoxide to form a potent nitrating and oxidizing radical called peroxynitrite (OONO), which has been found to play a significant role in pathophysiological alterations in IBD [13,14]. Indeed, iNOS expression levels and OONO levels have been shown to be significantly increased in patients with IBD [11,15]. Peroxynitrite has a very short half-life of 1.9 s at physiological pH and hence is very difficult to measure. Hence, 3-nitrotyrosine (3-NT) formation [14] or oxidation of dihydrorhodamine to rhodamine by OONO [16,17] are used to detect and measure OONO formation. In biopsy samples from IBD patients [10,18,19] and in animal models of chronic intestinal inflammation [20], 3-NT as a footprint for OONO production has been shown to be elevated.

In a rabbit model of IBD, as previously mentioned, unique changes in Na-alanine and Na-glutamine co-transport have been demonstrated. However, whether OONO may mediate these unique alterations in intestinal epithelial cells is not known. Therefore, the current study was undertaken to determine the effect of OONO on Na-amino acid co-transport in intestinal epithelial cells and determine the mechanism(s) of regulation of these co-transporters by peroxynitrite.

## 2. Results

### 2.1. Effect of OONO on Na-Alanine Co-Transport Activity in IEC-18 Cells

**Figure 1:**
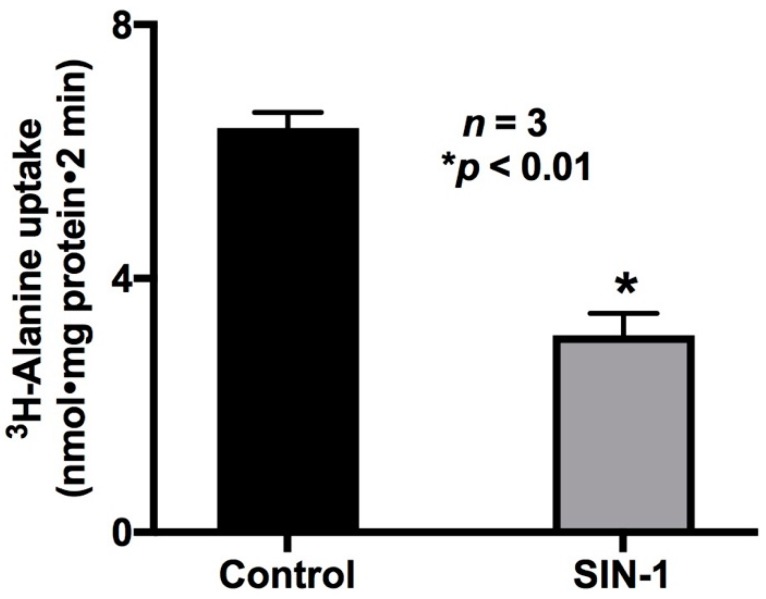
Effect of peroxynitrite (OONO) on Na-alanine co-transport (ASCT1) in IEC-18 cells. Na-stimulated ^3^H-alanine uptake was reduced in SIN-1-treated cells. Thus, OONO inhibits Na-alanine co-transport in IEC-18 cells.

SIN-1 treatment was used to demonstrate the effect of OONO on Na-alanine co-transport activity. SIN-1 chloride (amino-3-morpholinyl-1,2,3-oxadiazolium chloride), an active metabolite of molsidomine, produces both NO and superoxide under physiological conditions and therefore was used to generate OONO in vitro [21]. As shown in Figure 1, Na-alanine co-transport was significantly reduced by OONO in IEC-18 cells (6.4 ± 0.2 nmol/mg·protein/2 min in control and 3.1 ± 0.35 * nmol/mg·protein/2 min in SIN-1-treated cells; *n* = 3, * *p* < 0.01). These data demonstrated that Na-alanine co-transport was significantly inhibited by OONO.

### 2.2. Effect of OONO on Na/K-ATPase Activity in IEC-18 Cells

**Figure 2:**
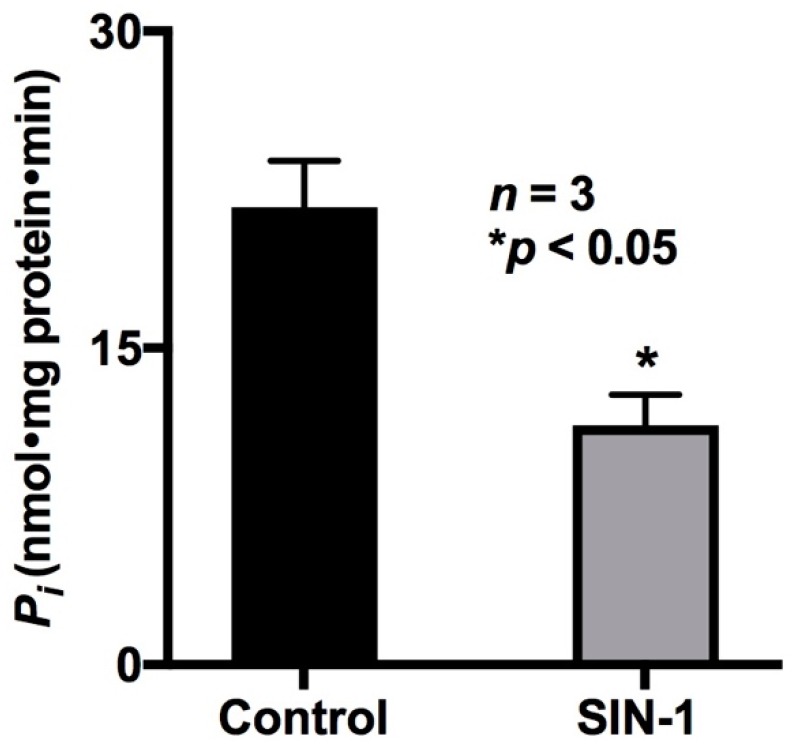
Effect of peroxynitrite on Na/K-ATPase activity in IEC-18 cells. Na/K-ATPase activity was measured as the amount of inorganic phosphate released in IEC-18 cell homogenates. Na/K-ATPase activity was found to be significantly decreased in IEC-18 cells treated with SIN-1 compared to control cells.

For secondary active transport processes such as ASCT1 and B0AT1, transcellular Na gradient is important for their proper functioning. Therefore, the effect of OONO production on Na/K-ATPase activity was studied in IEC-18 cells. As shown in Figure 2, SIN-1 treatment significantly reduced Na/K-ATPase activity (21.67 ± 2.16 and 11.33 ± 1.45 nmol/mg protein·min in control and SIN-1-treated cells, respectively; *n* = 3, * *p* < 0.05). Thus, the inhibition of ASCT1 by OONO may at least in part be secondary to altered transcellular sodium gradient in IEC-18 cells.

### 2.3. Kinetic Studies for the Inhibition of Na-Alanine Co-Transport Activity

**Figure 3:**
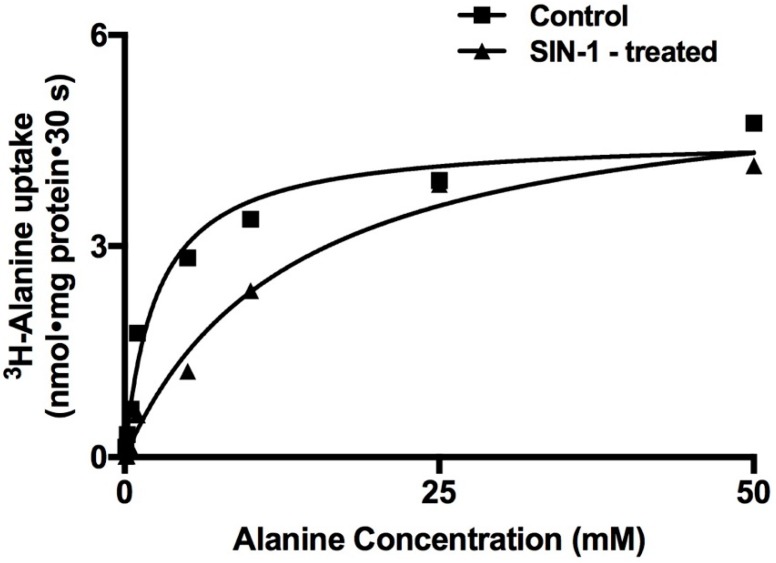
A representative graph of kinetics of Na-alanine co-transport in control and SIN-1-treated IEC-18 cells. Na-dependent ^3^H-alanine uptake is shown as a function of increasing concentrations of extracellular alanine at 30 s. As the concentration of extravesicular alanine was increased, uptake of ^3^H-alanine was stimulated and subsequently became saturated in both the experimental conditions.

To determine the mechanism of inhibition of Na-alanine co-transport by OONO, Na-dependent ^3^H-alanine uptakes were performed as a function of increasing concentrations of extra cellular alanine in IEC-18 cells. As the concentration of extra-cellular alanine was increased, the uptake of Na-dependent ^3^H-alanine was stimulated and subsequently became saturated in both the experimental conditions (Figure 3). Uptake data that were derived from these experiments were analyzed with GraphPad Prism 4 (GraphPad Software Inc., San Diego, CA, USA) for Michaelis–Menten kinetics (non-linear regression data analysis) to derive kinetic parameters *V_max_* and *K_m_*. Analysis of the kinetic parameters showed that the affinity (1/*K_m_*) for alanine uptake was significantly reduced by SIN-1 treatment (Table 1). However, the maximal rate of uptake (*V_max_*) of alanine was not affected in SIN-1-treated cells compared to control (Table 1). Thus, these data indicated that the mechanism of inhibition of ASCT1 by OONO was secondary to a reduction in the affinity of the co-transporter for alanine without an alteration in the number of co-transporters. 

### 2.4. Effect of OONO on ASCT1 Expression

**Figure 4:**
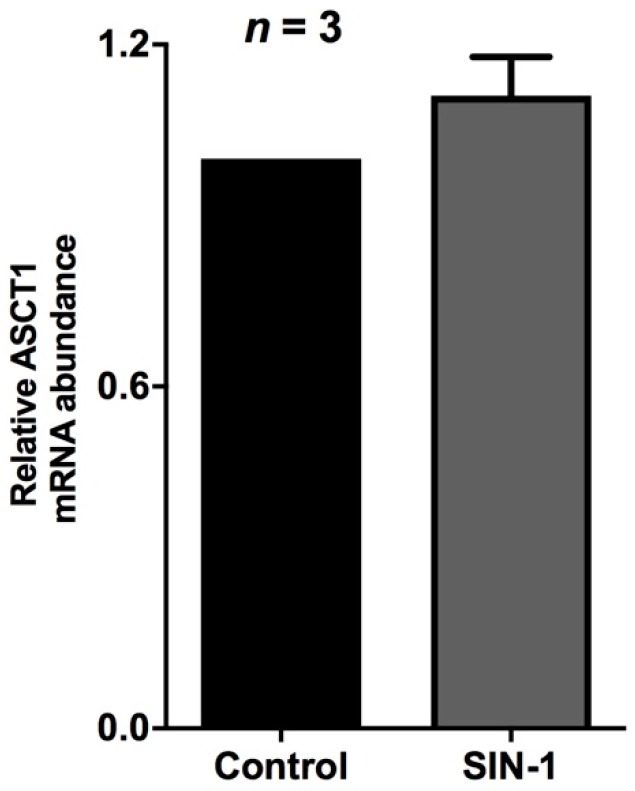
Effect of peroxynitrite on ASCT1 mRNA. Real-time PCR demonstrated that ASCT1 mRNA levels are unaffected by SIN-1 treatment in IEC-18 cells.

**Figure 5:**
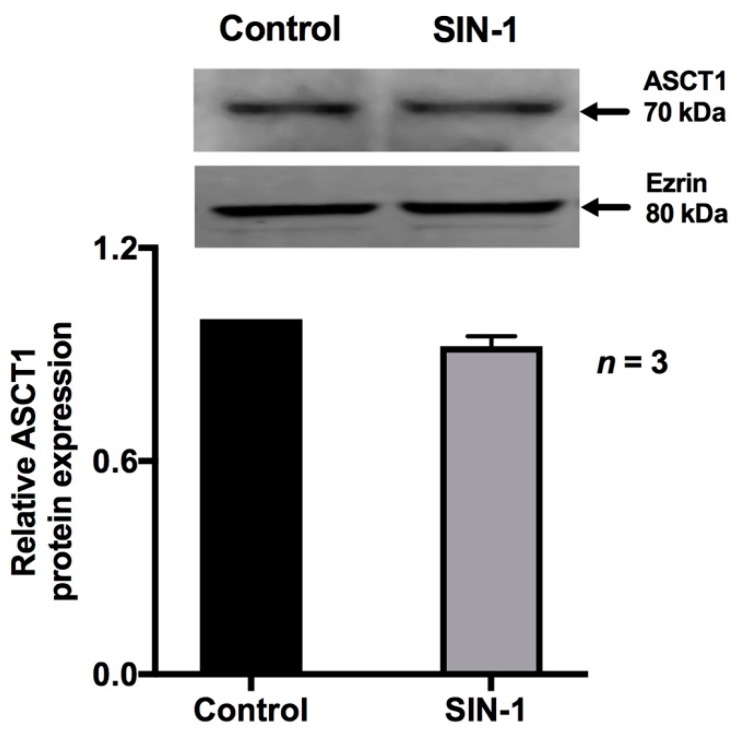
Effect of peroxynitrite on ASCT1 protein expression. A representative blot of ASCT1 protein expression that is unaffected by SIN-1 in IEC-18 cells is shown. Densitometric analysis showed that ASCT1 protein levels were unaltered by SIN-1 treatment compared to control.

ASCT1 is the predominant Na-alanine co-transporter in the BBM of IEC-18 cells. Real time PCR demonstrated that ASCT1 mRNA levels were unchanged in SIN-1-treated IEC-18 cells (Figure 4). Western blot studies also showed that SIN-1 treatment did not affect the immunoreactive protein levels of ASCT1 (Figure 5). As shown in Figure 5, densitometric quantitation substantiated these findings. These data, taken together with the data from kinetic studies, indicated that the mechanism of inhibition of ASCT1 by OONO was secondary to a reduction in the affinity of the co-transporter for alanine rather than an alteration in BBM co-transporter numbers.

### 2.5. Effect of OONO on Na-Glutamine Co-Transport Activity in IEC-18 Cells

**Figure 6:**
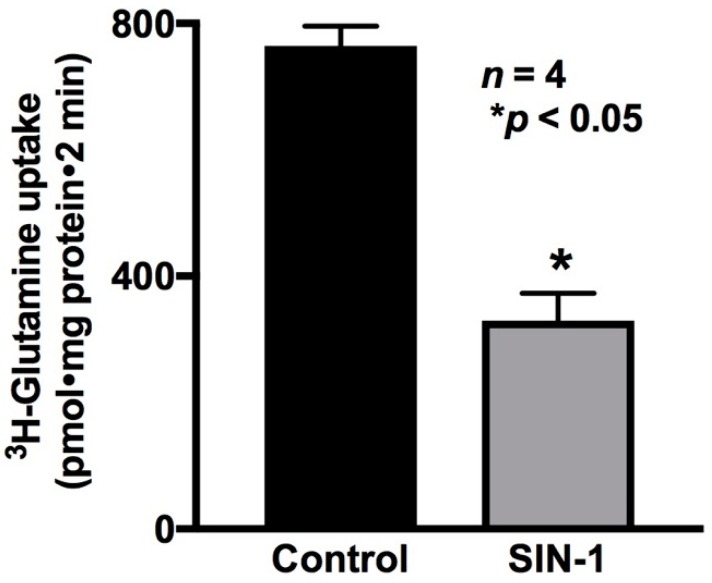
Effect of OONO on Na-glutamine co-transport in IEC-18 cells. Na-stimulated ^3^H-glutamine uptake was significantly reduced in SIN-1-treated IEC-18 cells. Thus, OONO inhibits Na-glutamine co-transport in IEC-18 cells.

The effect of peroxynitrite on a different Na-amino acid co-transport process, specifically, Na-glutamine co-transport in IEC-18 cells, was then determined. Na-stimulated ^3^H-glutamine uptake was measured as B0AT1 activity. As shown in Figure 6, Na-glutamine co-transport was significantly reduced by OONO formation in IEC-18 cells (764 ± 3 pmol/mg·protein/2 min in control and 329 ± 87 * pmol/mg·protein/2 min in SIN-1-treated cells, *n* = 3, * *p* < 0.05). These data demonstrated that Na-glutamine co-transport was significantly inhibited by OONO. 

### 2.6. Kinetic Studies for the Inhibition of Na-Glutamine Co-Transport Activity

**Figure 7:**
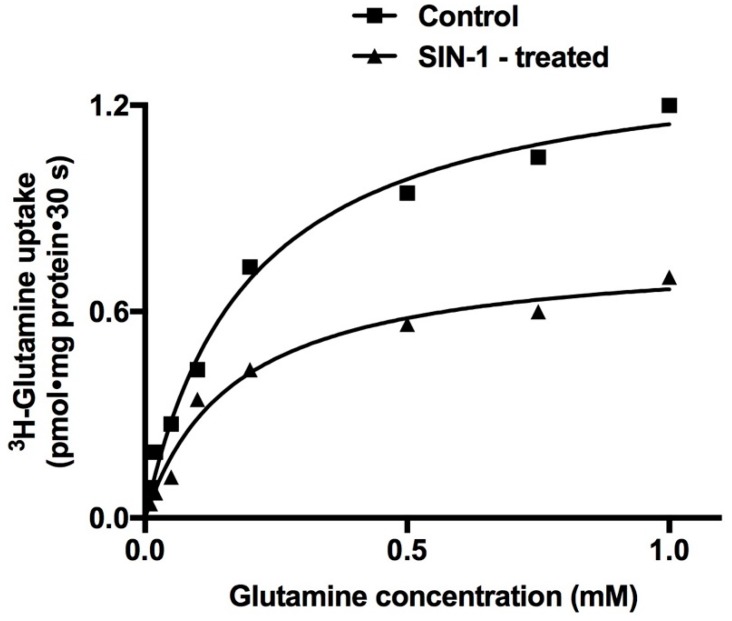
A representative graph of kinetics of Na-glutamine co-transport in control and SIN-1-treated IEC-18 cells. Na-dependent ^3^H-glutamine uptake is shown as a function of varying concentrations of extravesicular glutamine at 30 s. As the concentration of extravesicular glutamine was increased, uptake of ^3^H-glutamine was stimulated and subsequently became saturated in IEC-18 cells in both the experimental conditions. However, the maximal rate of uptake of glutamine (*V_max_*) was reduced by SIN-1 treatment, without a change in the affinity (1/*K_m_*) for glutamine uptake (Table 2).

Kinetic studies were performed to determine the mechanism of inhibition of B0AT1 by OONO formation in IEC-18 cells. Na-dependent ^3^H-glutamine uptake was performed at 30 s as a function of increasing concentrations of extra cellular glutamine in IEC-18 cells. As the concentration of extracellular glutamine was increased, the uptake of Na-dependent ^3^H-glutamine was stimulated and subsequently became saturated in both the experimental conditions (Figure 7). Uptake data that were derived from kinetic experiments were analyzed with GraphPad Prism 4 to derive kinetic parameters *V_max_* and *K_m_*. Analysis of the kinetics data by Michaelis–Menten non-linear regression analysis showed that SIN-1 treatment significantly reduced the maximal rate of uptake (*V_max_*) of glutamine, without changing the affinity (1/*K_m_*) for glutamine (Table 2). Thus, the kinetic data indicated that the mechanism of inhibition of B0AT1 by OONO was secondary to a reduction in the number of BBM co-transporters without altering the affinity of the co-transporter for glutamine.

### 2.7. Effect of OONO on B0AT1 Expression

**Figure 8:**
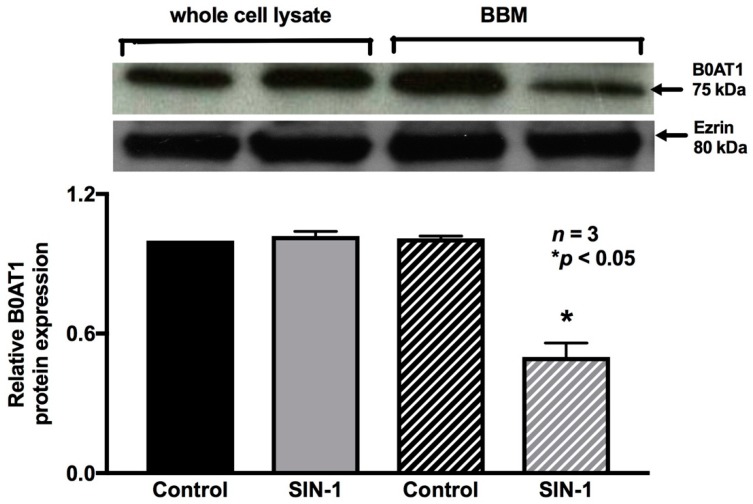
Effect of peroxynitrite on B0AT1 protein expression. In cellular homogenates, SIN-1 treatment did not affect the immunoreactive protein levels of B0AT1. However, in BBM, SIN-1 treatment did reduce the immunoreactive protein levels of B0AT1. Densitometry quantitation, as demonstrated with the graph, confirmed these findings.

B0AT1 mediates Na-glutamine absorption in IEC-18 cells. Therefore, the immunoreactive protein levels of B0AT1 were determined in the protein extracts of IEC-18 cells from both the experimental conditions. SIN-1 treatment did not affect the immunoreactive protein levels of B0AT1 in whole cell protein extracts (Figure 8), which was further confirmed by densitometric quantitation. However, these data are not consistent with kinetic studies demonstrated above, which indicated that the mechanism of inhibition of B0AT1 by OONO was secondary to a reduction in the BBM co-transporter numbers. Therefore, we performed Western blot studies of BBM protein extracts specifically. SIN-1 treatment did reduce the immunoreactive protein levels of B0AT1 in the BBM (Figure 8). Densitometric quantitation confirmed these findings. These data are consistent with kinetic studies, which indicated that the mechanism of inhibition of B0AT1 by OONO was secondary to a reduction in the BBM co-transporter numbers.

### 2.8. 3-Nitrotyrosine Formation

**Figure 9:**
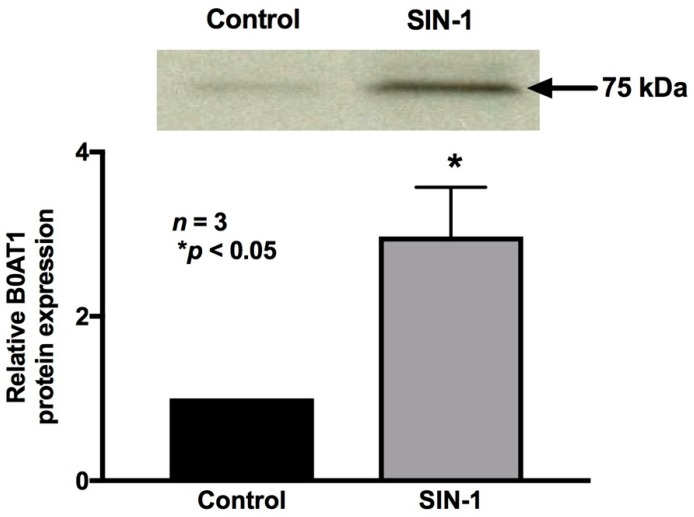
The effect of SIN-1 treatment on 3-nitrotyrosine formation in B0AT1 protein. Immunoprecipitated B0AT1 protein from SIN-1-treated IEC-18 cells showed an increased level of 3-nitrotyrisine compared to immunoprecipitated B0AT1 protein from control cells. Densitometric analysis showed that the 3-nitrotyrosine formation was significantly increased in B0AT1 immunoprecipitated from SIN-1-treated IEC-18 cells. These studies indicated that OONO modifies tyrosine residues of B0AT1 and thus inhibits its trafficking to the BBM and subsequently its transport activity in IEC-18 cells.

Unaltered protein levels of B0AT1 in cellular homogenates but reduced B0AT1 in BBM in SIN-1-treated IEC-18 cells indicates that the possible mechanism of alteration of B0AT1 by OONO may be at the level of trafficking from cytosol to BBM, specifically, the post-translational alteration of B0AT1 by OONO by 3-nitrotyrosine formation in B0AT1 protein. To determine the formation of 3-NT in B0AT1 in SIN-1-treated IEC-18 cells, we next performed immunoprecipitation analysis. The B0AT1 protein immunoprecipitated with B0AT1 antibody and immunoprobed for 3-nitrotyrosine, demonstrated clearly that B0AT1 is post-translationally modified by increased tyrosine nitration in SIN-1-treated samples (Figure 9). This was further substantiated by densitometric quantitation. These results provide evidence that OONO nitrates tyrosine residues of B0AT1, thus inhibiting its trafficking to the BBM and subsequently its transport activity in IEC-18 cells.

### 2.9. Uric Acid (UA) Reverses the Effect of OONO on ASCT1 and B0AT1 Activity 

**Figure 10:**
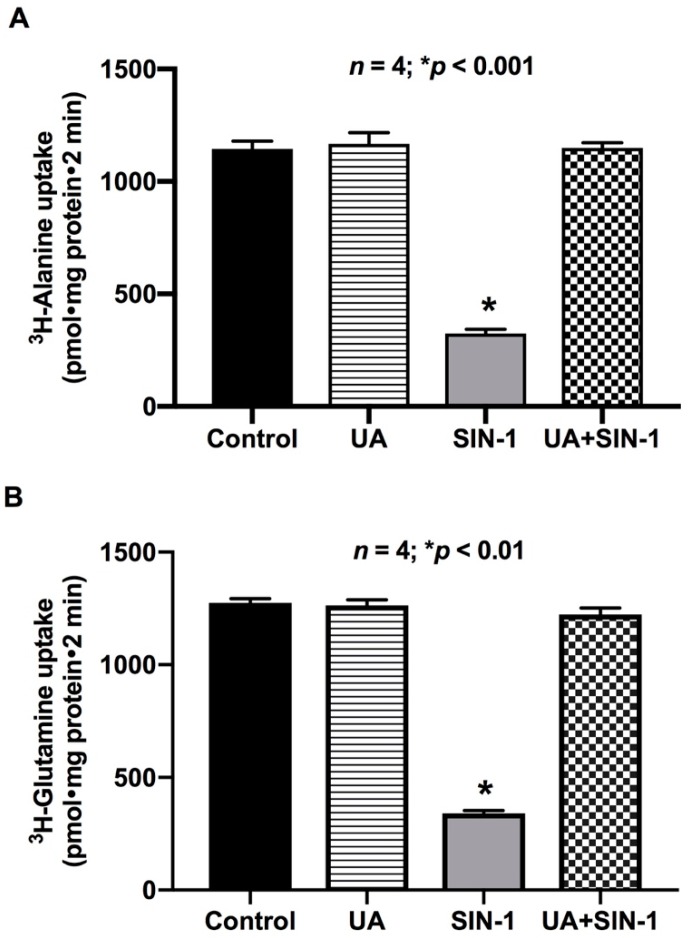
Uric acid (UA) prevents OONO effect on (**A**) ASCT1 and (**B**) B0AT1 activity. SIN-1 treatment significantly decreased ASCT1 and B0AT1 activities in IEC-18 cells. Uric acid (1 mM) pretreatment prevented the effect of SIN-1 treatment on both Na-amino acid co-transporters’ activities in IEC-18 cells. Thus, these data demonstrate that the inhibition of ASCT1 and B0AT1 activity was due to peroxynitrite produced from SIN-1 treatment.

Uric acid is a natural scavenger of cellular OONO, and therefore should prevent OONO-mediated ASCT1 and B0AT1 inhibition in IEC-18 cells. As shown in Figure 10, uric acid (1 mM) reversed the inhibition of ASCT1 and B0AT1 activities by OONO in IEC-18 cells. These data further indicate that OONO mediates alterations in ASCT1 and B0AT1 in SIN-1-treated cells. 

### 2.10. Nitric Oxide Levels in SIN-1-Treated Cells

**Figure 11:**
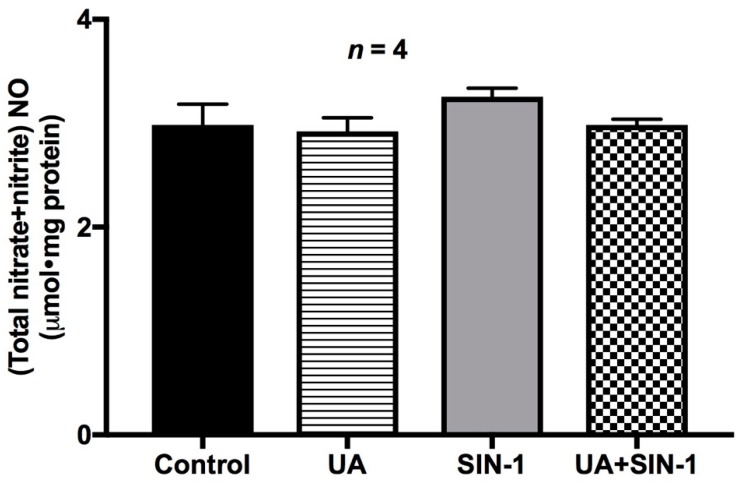
Nitric oxide levels in IEC-18 cells. Intracellular NO levels remained unchanged in SIN-1-treated cells and in cells treated with uric acid (UA) + SIN-1 compared to control IEC-18 cells, demonstrating that the altered effect seen in Na-amino acid co-transport by SIN-1 treatment is due to the combination of the nitric oxide and superoxide derivative OONO and not due to the intermediates alone.

SIN-1 produces both NO and superoxide, thereby generating peroxynitrite under physiological conditions. Inducible NO has also been shown to have deleterious effects on BBM Na-amino acid co-transport in the inflamed intestine [22]. Therefore, to confirm that the alterations seen with Na-amino acid co-transport in this study are indeed due to OONO formation by SIN-1 treatment and not due to its intermediates (NO or superoxide), more specifically not due to iNO, we determined the NO levels in SIN-1-treated cells. Physiological levels of NO were found to be unchanged in SIN-1 and uric acid-treated cells (Figure 11), confirming that the effect of SIN-1 on Na-amino acid co-transport was indeed due to OONO formation.

## 3. Discussion

Nitric oxide has a wide variety of effects in intestinal physiology in health and in disease states. In normal physiological state, constitutive nitric oxide (cNO) is known to have a regulatory role in the proper assimilation of nutrients and electrolytes in the intestine [23,24]. However, in pathophysiological conditions such as inflammatory bowel disease (IBD), where much larger levels of NO (known as inducible nitric oxide (iNO)) are produced, NO has deleterious effects including malabsorption of nutrients such as glucose and glutamine [22,25], the two most important nutrient sources for the intestine as well as the entire mammalian system. In addition to nutrient malabsorption, the significance of iNO-mediated tissue injury has also been well described during intestinal inflammation in IBD patients [10]. The deleterious effect of iNO is mediated indirectly when iNO combines to superoxide to form OONO, a well-known cytotoxic agent involved in numerous pathophysiological processes, and this has been demonstrated both in human samples as well as in animal model intestinal inflammation [26,27]. 

In this study, in vitro production of OONO in an intestinal epithelial cell line was found to affect two different amino acid absorptive mechanisms by two unique functional and molecular mechanisms. Na-alanine co-transport mediated predominantly by ASCT1 was inhibited secondary to a reduction in the affinity of the co-transporter for alanine. Na-glutamine co-transport mediated predominantly by B0AT1 was also inhibited similar to Na-alanine co-transport, however secondary to a reduction in BBM co-transporter numbers. The mechanism of inhibition of these two transporters are very similar to what was observed in a rabbit model of chronic intestinal inflammation, where ATB0 (the predominant Na-alanine co-transporter in the BBM of rabbit villus cells) was affected secondary to altered affinity and B0AT1 secondary to altered BBM co-transporter numbers [7,8,28]. In addition, the activity of Na/K-ATPase, which provides the driving force to these two amino acid absorptive pathways by maintaining optimum transcellular Na gradient, was also affected by OONO formation, very similar to the observations seen with the rabbit model of chronic intestinal inflammation. This demonstrates that the current study is in alignment with the in vivo observations obtained from the rabbit model of chronic intestinal inflammation, thus providing ample evidence that OONO generated in the inflamed mucosa may mediate malabsorption of amino acid absorption during chronic intestinal inflammation.

Uric acid, the product of purine metabolism, is one of the most physiologically important plasma antioxidants [29]. It is a strong scavenger of OONO, as demonstrated by its capacity to bind very specifically to OONO but not to NO [29,30,31,32]. In this study, IEC-18 cells were pretreated with uric acid to determine if the alterations seen with Na-amino acid co-transporters were indeed due to OONO produced by SIN-1 treatment. As demonstrated in the results, the counter effect of uric acid reversing the effects of OONO clearly indicated that the unique effects on ASCT1 and B0AT1 are indeed secondary to OONO production in the SIN-1-treated intestinal epithelial cells.

Peroxynitrite is an oxidizing and nitrating agent that reacts with a variety of biomolecules, including proteins and lipids [33,34]. One of the signature aftermaths of OONO formation is the nitrosylation of protein tyrosine residues, thereby affecting the target protein’s structure and function [35]. In this study, B0AT1 expression levels in the whole cell protein extracts were not altered by OONO treatment. However, the expression levels of B0AT1 protein at the BBM were significantly reduced in OONO-treated cells. This suggested that the trafficking of the B0AT1 protein from the cytosol to BBM, where the protein is functional, was altered. To determine if nitrosylation of B0AT1 tyrosine residues was a possible cause of this altered trafficking, B0AT1 protein was immunoprecipitated and its 3-nitrotyrosine levels were detected. It was found that in SIN-1-treated IEC-18 cells, B0AT1 3-nitrotyrosine level was significantly increased, compared to untreated cells. This revealed that the nitrosylation of B0AT1 protein altered its trafficking from cytosol to BBM, hence decreasing B0AT1 levels in BBM, thereby affecting its activity.

In summary, OONO uniquely affects Na-glutamine co-transport and Na-alanine co-transport in intestinal epithelial cells. Na-alanine co-transport mediated by ASCT1 was inhibited secondary to a reduction in the affinity of the co-transporter for alanine. Na-glutamine co-transport mediated by B0AT1 was also inhibited. However, the mechanism of inhibition was secondary to a reduction in BBM co-transporter numbers. More specifically, increased nitrosylation of this co-transporter seems to alter the cytosol to BBM trafficking of B0AT1. Further, at the intact-cell level, both were also inhibited secondary to diminished Na/K-ATPase. Finally, uric acid, a scavenger of OONO, reverses the effects of OONO, clearly indicating that the unique effects on ASCT1 and B0AT1 are indeed secondary to OONO. Thus, both at the cellular level as well as at the level of the co-transporter in the BBM, OONO, in vitro, affects Na-alanine and Na-glutamine co-transport identically to how they are affected in vivo in the chronically inflamed rabbit intestine.

## 4. Materials and Methods

### 4.1. Tissue Culture and Drug Treatment

IEC-18 cells (ATCC CRL-1589, American Type Culture Collection, Manassas, VA, USA) were grown with high glucose Dulbecco’s modified Eagle’s medium supplemented with 2 U/mL of insulin, 0.5 mM β-hydroxybutyrate, and 10% fetal calf serum. The cells were grown at 37 °C with 10% CO_2_. For OONO production, IEC-18 cells were treated with SIN-1 chloride (OONO donor; 100 μM) for 24 h. Control cells were treated with the vehicle.

### 4.2. Na-Amino Acid Co-Transport in IEC-18 Cells

Na-dependent amino acid uptake experiments were performed on IEC-18 cells plated on 24-well plates, as previously described [25]. Uptake experiments were performed at desired time intervals in reaction medium containing either 130 mM NaCl or 130 mM TMACl in HEPES medium with 10 µCi of ^3^H-alanine and 100 µM cold alanine for ASCT1 or ^3^H-glutamine and 100 µM cold glutamine for B0AT1. The uptakes were stopped by the addition of cold Na-free buffer, after which the cells were washed once with the same ice-cold buffer. The cells were then extracted from the plates by incubation with 1 N NaOH for 20 min at 70 °C. A 4 mL portion of scintillation fluid (Ecoscint; National Diagnostics, Atlanta, GA, USA) was added to each of the reaction extracts and the radioactivity was determined in a Beckman 6500 Beta scintillation counter (Beckman Coulter Inc., Brea, CA, USA).

### 4.3. Na/K-ATPase Assay

Na/K-ATPase activity was measured on IEC-18 cellular homogenates as previously described [23,36]. Na/K-ATPase activity was measured as the amount of inorganic phosphate released, which was expressed as nanomoles/milligram protein/minute.

### 4.4. Molecular Studies

Brush border membrane (BBM) for Western blot analysis was prepared from IEC-18 cells using a protocol that was described before [37]. Protein extracts from the BBM and total cell lysate of IEC-18 cells were prepared and analyzed by Western blot using previously used standard protocols [25]. In this study, rat ASCT1 and rat B0AT1 specific antibodies and secondary antibodies coupled to horseradish peroxidase, all obtained from Abcam (Abcam PLC, Cambridge, MA, USA), were used to detect the respective proteins. Ezrin was used as the loading control in all the Western blot experiments. Immunoprecipitation with rat B0AT1-specific antibody and Western blot with anti-3-nitrotyrosine antibody was done to analyze nitrosylation levels of B0AT1 protein from BBM. The protein density of the specific proteins was quantitated with a densitometric scanner FluorChemTM instrument (Alpha Innotech, San Leandro, CA, USA). Real-time quantitative PCR was performed with rat ASCT1-specific primers and probe (ThermoFisher Scientific Inc., Waltham, MA, USA). *ASCT1* gene expression was standardized against β-actin expression for each of the experimental conditions.

### 4.5. Measurement of NO

IEC-18 cellular homogenates were prepared and used for total nitric oxide measurement by a protocol described previously [38]. Griess reaction colorimetric assay kit (Item No. 780001, Cayman Chemical Company, Ann Arbor, MI, USA) was used to measure total NO levels in cellular homogenates from different experimental conditions. A nitrate standard curve was generated to determine NO levels in the cellular homogenates at 540 nm.

### 4.6. Statistics

The results of the data presented in this study represent means ± SEM of the experiments performed in triplicate. Student’s *t*-test was performed using GraphPad Instat 4 (GraphPad Software Inc., San Diego, CA, USA) to determine statistical significance for the data generated for the different experimental conditions.

## 5. Conclusions

In conclusion, both Na-alanine and Na-glutamine co-transporters are regulated by OONO in intestinal epithelial cells by mechanisms similar to those seen in villus cells from the rabbit model of chronic intestinal inflammation. Moreover, this study provided the additional mechanistic information on the OONO-mediated regulation of Na-amino acid co-transporters by OONO, which is known to be increased in the inflamed mucosa during chronic intestinal inflammation. The novel findings of this study may provide useful therapeutic options to counteract nutrient malabsorption that is known to occur in patients with IBD.

## Figures and Tables

**Table 1:** Effect of SIN-1 treatment of the kinetics of Na-alanine co-transport in IEC-18 cells. The *V_max_* of Na-alanine co-transport was unaffected by SIN-1 treatment, but the affinity (1/*K_m_*) for alanine uptake was reduced in the SIN-1-treated IEC-18 cells (*n* = 3; * *p* < 0.01).

	*V_max_* (µmol/mg Protein·30 s)	*K_m_* (mM)
Control	4.5 ± 0.3	2.5 ± 0.3
SIN-1	5.4 ± 0.8	13.3 ± 0.5 *

**Table 2:** Kinetic parameters of Na-glutamine co-transport in IEC-18 cells. The *V_max_* of Na-glutamine co-transport was decreased by SIN-1 treatment, but the affinity (1/*K_m_*) for glutamine uptake remained unchanged by SIN-1 treatment in IEC-18 cells compared to control (*n* = 3; * *p* < 0.05).

	*V_max_*(pmol/mg Protein·30 s)	*K_m_* (mM)
Control	1.48 ± 0.06	0.18 ± 0.01
SIN-1	0.76 ± 0.01 *	0.17 ± 0.004
